# Professional Roles in Danish Clinics for General Late Effects After Cancer—A Qualitative Study

**DOI:** 10.1007/s13187-025-02660-9

**Published:** 2025-06-06

**Authors:** K. B. Dieperink, R. Skøtt, T. Mattsson, L. B. Thisted, C.Ø. Christensen, L. K. Tolstrup

**Affiliations:** 1 Department of Clinical Research, University of Southern Denmark, Campusvej 55, 5230 Odense M, Denmark; 2 Department of Oncology, Odense University Hospital, Sdr. Boulevard 29, 5000 Odense C, Denmark; 3 Department of Oncology, Zealand University Hospital, Vestermarksvej 9.2, 4000 Roskilde, Denmark; 4 Department of Oncology, Hospital Sønderjylland, Sydvang 1, 6400 Sønderborg, Denmark

**Keywords:** Interdisciplinarity, Late effect clinics, Nurse’s roles, Psychodynamics, Physician roles, Psychologist’s roles, Survivorship clinics

## Abstract

Late effect clinics (LECs) post-primary cancer treatment emerge. We explored healthcare professionals’ experiences working in Danish LECs from a psychodynamic perspective seeking to understand how professionals from different disciplines navigate their roles and collaborate within the context of survivorship care. We employed a qualitative approach through three virtual focus groups with nurses, physicians, and psychologists *n* = 15. All respectively working in five different LECs with patients and caregivers affected by general late effects after cancer treatment. Data were analyzed using thematic analysis. Four themes were found: Transformation towards person-centered care, therapeutic space as a precondition, redefined professional boundaries, and challenges due to traditional hospital structures. The themes revealed that patients with late effects and their caregivers require a bio-psychosocial and person-centered approach, with an emphasis on creating a therapeutic space as a prerequisite for cooperation. The professionals were willing to expand their professional boundaries but were challenged by systemic hierarchy and the constant need for competency and team development. Experiences and dynamics within professional roles in Danish LECs have significant implications for rethinking the organization and professional development of care. These insights suggest the need to empower healthcare professionals, adapt to evolving professional roles, and enhance person-centered care in this unique survivorship context. Improved collaboration across roles could provide survivors with more cohesive, supportive care, though traditional hospital hierarchies pose challenges. Increased professional development promises a higher level of tailored, competent care for addressing complex late effects during survivorship.

**Supplementary Information:**

The online version contains supplementary material available at 10.1007/s13187-025-02660-9.

## Background

In Europe, there is a notable increase in cancer survivors due to several factors: increasing cancer incidence due to an aging population, earlier detection of cancer, and significant advancements in cancer treatments [1]. However, this trend exposes shortcomings in existing care models, which struggle to address the complex array of late effects, encompassing physical, psychosocial, and supportive care needs post-cancer treatment [2].

In Denmark, more than 50% of cancer survivors encounter one or more late effects from their cancer and/or cancer treatment, adversely affecting their quality of life [3]. Many of these patients experience that these late effects are being neglected as conventional cancer follow-up protocols primarily focus on monitoring for relapse and lack the multidisciplinary approach necessary to manage complex late effects, resulting in inconsistent and insufficient quality of care for survivors [4]. Thus, current models of care are unsustainable and fail to address the many unmet needs of survivors of cancer [5]. In the USA, the National Comprehensive Cancer Network recommends routine provision of survivorship care after treatment of adult-onset cancer [6]. A national survey that examined the prevalence, types, and outcomes of cancer survivorship services at accredited facilities, providing cancer care to three-quarters of all US patients with cancer, found that over 80% of institutions considered their survivorship services as beneficial, yet reported that only a minority received them [7]. Similarly, the ESMO Expert Consensus Statements on Cancer Survivorship advocate for the implementation of a comprehensive framework to enhance survivorship care [8]. In line with this recommendation, the Region of Southern Denmark established four late effect clinics (LECs) in 2022. These clinics can be compared with survivorship clinics in the USA. Additionally, the Region of Zealand launched a similar clinic. Despite the significant demand, only two out of Denmark’s five regions have implemented such clinics, likely due to resource constraints and differing regional priorities.

The clinics aim to centralize care for adult cancer survivors with complex late effects. Collaboration between the LECs in the two regions facilitates streamlined patient pathways, weekly online multidisciplinary team (MDT) meetings discussing difficult patient cases, uniform data collection including collection of patient-reported outcomes, and the development of treatment protocols.

The workflow in LECs differs significantly from traditional treatment clinics because survivors with late effects often present complex symptom clusters [9], with no clear treatment guidelines available addressing these clusters. We are only beginning to understand the physical, mental, and social challenges faced by cancer survivors with late effects and their families [10, 11]. Many of these late effects reflect a complex and interrelated picture with frequent symptoms of fatigue, sleep disturbances, cognitive impairment, pain, fear of recurrence, and family and work-related challenges, for instance, in relation to retention in the labor market [12].

Addressing these challenges requires an interdisciplinary and holistic approach, yet many healthcare professionals lack formal education in survivorship care, necessitating adaptation and integration of their skills into this new context. Despite the relevance and importance of LECs, there is limited research on healthcare professionals’ experiences in this setting, particularly regarding their perceptions of working in interdisciplinary teams. A recent scoping review by Phothikul and Seven found 14 studies exploring knowledge, perceptions, skills, and practices among oncology nurses providing survivorship care. The study demonstrated limited available research, with a specific gap in integrating knowledge into survivorship practices [13]. Thus, there is a notable lack of research on how various professional groups perceive working in survivorship care, particularly regarding the collaboration among them. Psychodynamic theory offers a way to explore the unconscious emotions, conflicts, and defense mechanisms that influence healthcare professionals’ behaviors and collaboration. While this perspective has been applied in areas such as oncology and palliative care to understand emotional labor and professional dynamics [14, 15], it remains underexplored in survivorship care. By using a psychodynamic framework, this study addresses a gap in existing research by examining how unconscious dynamics shape professional roles, boundaries, and interdisciplinary teamwork in late effect clinics.

## Aims

This qualitative study aimed to explore Danish healthcare professionals’ experiences working in LECs from a psychodynamic perspective, seeking to understand how professionals from different disciplines navigate their roles and collaborate within the context of survivorship care for patients and caregivers affected by late effects after cancer and its treatment.

## Methods

### Design

The study employed a qualitative approach through focus groups [16, 17], drawing upon a psychodynamic framework. Psychodynamics is a psychological theory investigating the interplay between the unconscious mind and conscious thoughts and behaviors. It seeks to comprehend the psychological forces shaping human behavior, emotions, thoughts, and fantasies [18]. The focus groups aimed to explore how psychodynamic processes manifest within three different groups of health professionals and how these dynamics influence group cohesion and performance. By examining the emotions, potential conflicts, and defense mechanisms within their roles, we sought to gain insights into how nurses, physicians, and psychologists in five Danish LECs manage their professional identities while working collaboratively in survivorship care. The Standards for Reporting Qualitative Research (SPQR) was used as a reporting guideline [19].

### Data Collection

Participants were recruited from a total of 20 professionals currently employed across five Danish LECs in the Regions of Southern Denmark and Zealand. We had access to a mail list with all professionals included, and all were invited. Data were collected through video focus groups. Due to the exploratory nature of the study, the sample size could not be precisely determined a priori, but to reach data saturation, we aimed to include approximately 15–20 professionals with three different educational backgrounds: nurses, physicians, and psychologists.

### Inclusion Criteria

- Aged 18 years and older
- Working in a Danish general LEC
- Danish speaking

### Study Procedures

We conducted three focus groups over video using Webex; one with nurses, one with physicians, and one with psychologists. We used video connection as this was the normal communication tool among the included professionals to eliminate time waste, i.e., travel. An interview guide was developed to ensure framing consistency (supplementary file), and interviews were carried out by the same interviewer, RS. The semi-structured interview guide was based on information extracted from existing literature on the topic. The development of the interview guide was also informed by the psychodynamic framework, with questions designed to explore not only practical and organizational aspects of working in LECs but also unconscious dynamics such as emotional reactions, role boundaries, professional identity, and interprofessional group dynamics. The interviews covered topics related to uncovering the psychodynamic interplay between the health professionals.

All interviews were video recorded and transcribed verbatim. The interviews took an average of 54 min (range 52–56 min).

In addition, we collected relevant healthcare professionals’ characteristics: age, gender, education, years since education, years in oncology, and months employed in the LEC (Table 1).

**Table 1:** Participant characteristics, *n* = 15

Age	
Years, mean (SD)	48 (8.0)
Range	32–58
Employed at cancer departments *n* (%)	
Odense University Hospital	4 (26.67)
Sønderborg Hospital	4 (26.67)
Vejle/Esbjerg Hospital	3 (20.0)
Zealand University Hospital	4 (26.67)
Educational background *n* (%)	
Nurse	8 (53.33)
Physician	5 (33.33)
Psychologist	2 (13.33)
Educational attainment *n* (%)	
Bachelor	2 (13.33)
Master	6 (40.00)
PhD	7 (46.67)
Healthcare professional experience	
Years, mean (SD)	21 (10.2)
Range	6–34
Oncology experience	
Years, mean (SD)	14 (7.6)
Range	2–32
Late effect clinic experience	
Months, mean (SD)	20 (3.3)
Range	14–24

### Analyses

Thematic analysis was applied to the qualitative data to identify, report, and categorize patterns into themes [20]. Thematic analysis is a systematic approach comprising six steps: (1) familiarization with data, (2) generating initial codes, (3) searching for themes among codes, (4) reviewing themes, (5) defining and naming themes, and (6) producing the final report [20]. After data condensation, the video data were revisited with a specific focus on identifying non-verbal cues, such as facial expressions, body language, and tone of voice, emotional change or recurring statements (repetition compulsion), and moments where verbal content appeared to contrast with the participant’s emotional expression or behavior. Discrepancies were noted when, for example, a participant verbally conveyed certainty while showing physical signs of discomfort, e.g., nervous laughter, avoiding eye contact, or visible tension. These observations were documented alongside the transcript data and interpreted within the psychodynamic framework to uncover unconscious dynamics, emotional defenses, and relational patterns influencing professional roles and teamwork. This interpretative process was integrated with the thematic analysis, contributing to a comprehensive understanding of the psychodynamics at play in the interdisciplinary LEC setting.

The interviewer RS conducted the initial analysis, which was subsequently validated by KBD and LKT. The interviewer RS was skilled in qualitative research, psychodynamic interviewing, and an experienced cancer nurse, but had no experience working in a LEC. Thus, he had no preconceived notions.

### Ethics

According to Danish law, approval from the ethical committee was not required, but the study was registered with the Danish Protection Agency, Acadre no: 23/37575. All participants received written and verbal information and provided written consent for participation. The study was conducted following the Declaration of Helsinki [21]. Data were secured in SharePoint.

## Results

### Participants

In September 2023, 20 healthcare professionals were contacted by mail and asked to participate. Of these, five declined due to work obligations. The 15 enrolled participants were nurses (*n* = 8), physicians (*n* = 5), and psychologists (*n* = 2). All were female. The mean age of participants was 48 years (SD 8.0); (range 32–58). Participants were experienced in general healthcare and oncology specifically, but had less than 2 years of experience working in a LEC. Several had master’s or PhD degrees (Table 1). It was characteristic of the participants that each member had their own specific professional interest areas or further education, which they also had the opportunity to pursue in their employment at a LEC. For instance, a nurse was also trained as a sexologist, and a doctor was trained as a psychotherapist. Several nurses specialized in fatigue, sleep, and energy management, while the psychologists focused particularly on fear of recurrence and identity loss.

### Themes

**Fig. 1:**
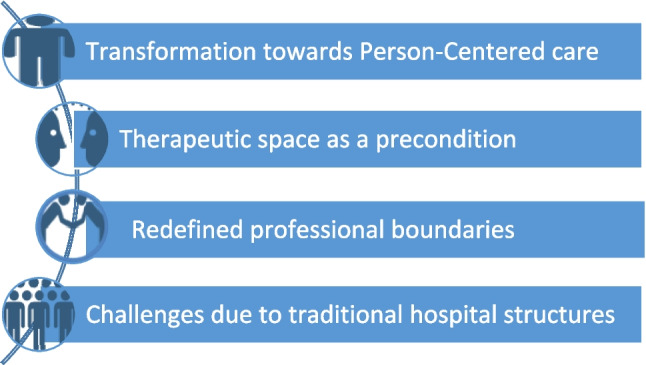
Themes of a qualitative study of professional roles in a late effect clinic

We extracted four themes from the three focus groups: transformation towards person-centered care, therapeutic space as a precondition, redefined professional boundaries, and challenges due to traditional hospital structures (Fig. 1).

Table 2 presents representative key excerpts to reflect the themes. In the following, we describe the emerged psychodynamic themes.

**Table 2:** Themes with representative key excerpts and psychodynamic analysis

Theme	Quote	Psychodynamic analysis
Theme 1: Transformation towards person-centered care	“[…] It’s a completely different approach you have with the patients—but a space you’re curious about, but also a bit uncertain about.” (nurse)	Describing the unique approach within LEC, this quote captures the curiosity and uncertainty healthcare professionals may experience as they engage in more person-centered care
	“You develop a completely different relationship with the patients in the LEC, because you gain a completely different understanding of them, and we make an agreement with them (the patients) and ourselves that together we will help them” (physician)	Describing the contrast in the relationship and a new partnership working with patients in a LEC instead of treating patients in a cancer clinic. The mentioning of making an agreement with the patients points to a strong therapeutic alliance. This partnership approach can empower patients, making them active participants in their healing process
	“I increasingly experience that it’s a process because there’s no quick fix—but it’s very much a process in their lives.” (nurse)	Acknowledging the complexity of patient journeys, this quote emphasizes the continuous, transformative process experienced by patients within the context of LECs
	“We cover a lot of ground. The patients appreciate that we are willing to listen to their issues and their life stories. I don’t promise them anything in advance. It may also be about learning to live with certain things, but that can also help the patient” (physician)	Understanding that listening and accepting conditions is an important focus in a LEC. By not promising anything in advance, the physician might be managing transference, where patients project feelings and expectations from past relationships onto the health professional. By not offering quick solutions and instead focusing on the exploration of issues, the physician may be aiming to facilitate deeper insight
	“The conversations take a bit longer because we need to cover a lot of ground, and the charting work also takes longer, at least for me, as I put extra effort into describing the general symptom picture to help our doctors and nurses as much as possible. I have the impression that they also spend more time reading my notes for LEC patients than they do for ‘regular’ patients.” (psychologist)	Describing the change of workflow based on the different patient needs but also considering the team as a recipient of information for the further patient trajectory. The distinction between LEC patients and regular patients could imply more complexity that necessitates a more intensive and detailed individualized approach
	“LEC patients are interesting, even though they are complex. They just take more time and involve more paperwork. Often, they are bounced around in the job market and need status statements. The additional work and interdisciplinary collaboration are more significant with these patients.” (psychologist)	This quote emphasizes the intricate nature of working with LEC patients and underscores the vital role of collaborating with other professionals to ensure success
	“Whether we want it or not, we have more time, but here is another opportunity to dig deeper.” (physician)	Recognizing the temporal aspects of patient interactions, this quote suggests the potential for a more profound exploration of patient needs, contributing to the transformative nature of healthcare delivery within LEC
Theme 2: The therapeutic space as a pre-condition	“We have greatly benefited from having the psychologist close by, who has challenged us on the ‘medical’ aspect. Being in the moment, you don’t necessarily have to solve things. You just have to listen. This is very different from our usual doctor role, and can it be therapeutic in itself? Yes, it can.” (physician)	Recognizing that other professionals contribute with essential skills necessary for achieving our common goal
	“It’s the psychosocial nursing care that really comes into play, something that requires new competencies and constant learning—that’s why the national network is so important.” (nurse)	Emphasizing the evolving nature of nursing care, this quote underlines the importance of continuous learning and new competencies, highlighting the dynamic and transformative aspects of the nursing role within the therapeutic space of LEC
	“I think we can call a lot of our conversations therapeutic—not that we play psychologists, but… but we can certainly call a lot of our conversations therapeutic.” (nurse)	Describing the therapeutic nature of interactions, this quote underlines how nurses, while not psychologists, contribute significantly to the emotional and psychological well-being of patients within LEC
	“Our role is especially regarding the individual patient, but also interdisciplinary, to provide colleagues with a greater understanding of the correlation. How the mind affects the body, and the body affects the mind.” (psychologist)	The psychologists see themselves as important for competence development in the team, and experience acknowledgement of their specific skills
	“Some patients might benefit from seeing a psychologist earlier in the cancer treatment process so that they wouldn’t end up becoming LEC patients.” (psychologist)	This statement suggests that early psychological intervention in the cancer trajectory might prevent some patients from developing late effects. It underlines the potential preventative role that psychologists can play. Meanwhile, it may also highlight a feeling of insecurity in the professional role
	“Competence development has a significant impact on qualifying the work we do, which involves such a complex interplay between the psychological and somatic that it is almost impossible to see where one ends and the other begins, or vice versa.” (psychologist)	Underlines the necessity of continuous professional development to navigate the intricate and intertwined nature of psychological and somatic health in patient care
Theme 3: Redefined professional boundaries	“Doctors, psychologists, and sexologists all have clearly defined roles—and they can do things that I simply can’t—I, as a nurse, am more generalized.” (nurse)	Articulating the distinct roles among healthcare professionals, this quote underscores the specialized expertise of other professionals and emphasizes the generalized nature of the nursing role within LEC which can also be a challenge
	“The nurses indeed have unique skills and abilities that I don’t have, so it’s not that the doctor knows best in this situation—not at all. It’s precisely that we have different competencies. I don’t necessarily know more than the nurses and psychologists. We just have different roles in this.” (physician)	Recognizes the importance of a team-based approach in healthcare, where the unique contributions of each professional are essential for achieving the best patient outcomes. The physician’s acknowledgment that they do not have the same skills as nurses and psychologists reflects humility and self-awareness. In psychodynamic terms, this could indicate that the physician’s secure sense of self, allows them to recognize and accept their limitations without feeling threatened
	“Here, I have at least felt a kind of… performance pressure, a little… a bit of pressure like: oh, what does the patient expect from me; and can I do it, and am I stepping into an area I’m not trained for… now I also have to make sure to stay on the path.” (nurse)	Conveying the nuanced pressures faced by healthcare professionals, this quote highlights the dynamic nature of their roles and the need to navigate patient expectations, potentially influencing the transformative nature of their interventions
	“The nurses are coordinators for all the cases, so I am deeply reliant on them also having the time to manage those cases. I see us as a team, and we are equals who can consult with each other. We each bring different skills to the table.” (physician)	Appreciation for the essential role of nurses in patient care coordination, the importance of teamwork, and the value of diverse professional skills in providing comprehensive healthcare
	“Sometimes they (nurses) can handle certain things because they know there isn’t time, or it’s not extensive enough to require a psychologist.” (psychologist)	Signifying the collaborative approach to patient care, this quote underlines how nurses contribute to a comprehensive understanding of patients’ conditions before other specialists further elaborate on the intricacies
	“In the beginning, I thought I needed a psychotherapy course because I felt out of my comfort zone, but I have gradually realized that it is not so daunting. I think it is a privilege that we have the time to understand what is going on behind and around the patient, and I take that knowledge with me into my regular clinic”. (physician)	From discomfort to confidence and the value of a holistic understanding of patients, and the integration of psychological insights into regular medical practice
	“Nurses might not feel confident stepping into the psychological field if they didn’t have extensive experience in the cancer field. However, I think they are quick to speak up when the psychological aspect becomes too intense, and when there are deeper patterns, and then they are good at saying it should be referred to a psychologist.” (psychologist)	Highlights the importance of experience in building confidence, the proactive role of nurses in recognizing the need for psychological referrals, and the effective teamwork required to provide care for a patient in LEC. Therefore, it can also contain an unconscious fear of unclear roles or overstepping of role boundaries that would compromise this teamwork
	“I also experience that…—I would call it more of a coordinator role—and I don’t necessarily feel that we coordinate everything, but we have the patient’s pathway in our hands.” (nurse)	Describing a nuanced coordinator role, this quote highlights the redefined boundaries and responsibilities of nurses, particularly in shaping patient pathways within the therapeutic space of LEC
	“Experienced nurses are very valuable, but we have the knowledge of various specialties and differential diagnoses, so the responsibility falls on us.” (physician)	The distribution of responsibilities is different in the team. The ultimate responsibility for diagnosis and treatment falls on the physicians. This is a structural and legal framework that strains the cohesion of the team as power is unevenly distributed
	“I can sometimes feel that I don’t have a fixed position as a nurse in LEC—I think that can be difficult sometimes.” (nurse)	Acknowledging the challenges of role ambiguity, this quote reflects the uncertainties nurses may face in establishing a fixed professional position within the evolving therapeutic space of LEC
	“I hope we work interdisciplinarily. I know that we sometimes work in parallel disciplines, but we also work across disciplines. I would like to take on some tasks and be a leader in that, but I also think the other professional groups should take on tasks and lead them.” (physician)	Reflects a commitment to interdisciplinary collaboration and a desire for balanced leadership and task sharing within the healthcare team. The belief that other professional groups should also take on tasks and lead them shows respect for the competencies and leadership potential of all team members
	“There are certainly cases I don’t need to handle. It’s been about fatigue or fear of relapse, which they (nurses and psychologists) can manage just fine. But as soon as it involves pain, urinary issues, bowel problems, or swallowing difficulties, I definitely get involved.” (physician)	To prioritize involvement based on the complexity of medical issues, while recognizing the role of other professionals in managing less complex problems or concerns with non-medical treatment
Theme 4: Challenges due to traditional hospital structures	“Traditionally the ‘doctor’ was the clinic, and now we are involving more professional groups, so I feel like I am the clinic. Nurses are being involved, but it is definitely me who decides what should happen. They are allowed to do what they are permitted to do, but it is clearly me who directs things” (physician)	Reflects the transition from a traditional, physician-centric model of care to a more collaborative, multidisciplinary approach while still emphasizing the physician’s continued sense of primary responsibility and authority in directing patient care
	“In some way, it’s the doctors who set the agenda for our Multidisciplinary Team Conferences—and it’s not because I want to accuse anyone—but we [the nurses] are not good at taking it and saying: Let’s take a new professional perspective perhaps.” (nurse)	Revealing power dynamics, this quote illustrates challenges in advocating for alternative professional perspectives within the multidisciplinary team, pointing to potential impediments to effective collaboration
	“Our doctor is definitely the primary coordinator with us. We just don’t have the necessary time to consult with each other.” (psychologist)	Reflects the importance of the physician’s role and the challenge of limited time for interprofessional consultation. It underlines a need for better communication strategies to enhance teamwork and improve patient care
	“I feel like I’m caught in a sort of half-position. It’s not only about what you’re authorized to do by your superior but also what you’re empowered to do from below. You end up in a middle role that can be hard to define. It becomes a kind of pseudo-leadership function. Being a semi-leader due to one’s professional expertise, because doctors often find themselves in leadership roles more than others, is a challenging role.” (physician)	It highlights the difficulties and challenges associated with being in a semi-leadership position, shaped by both professional expertise and the dynamics of medical practice. The concept of a “pseudo-leadership function” could reflect the physician’s struggle with their professional identity. They might feel torn between being a colleague and a leader, creating an internal conflict about their roles and self-perception within the team
	“In LEC, the nurse also has a half-secretarial function—which I don’t find very exciting, to be honest.” (nurse)	Expressing discontent with certain administrative responsibilities, this quote touches upon the challenges nurses face in balancing therapeutic roles with administrative tasks, often placed by nurses as part of the traditional hierarchy
	“I actually don’t know if it’s the doctor with us who acts as a kind of leader of the LEC. It might be, but I’m not entirely sure. I don’t think they view it in such a hierarchical way. It’s as if we are all completely on the same level, although I would say that the two nurses we have handle a lot, coordinate a lot, see many patients, and manage a whole lot.” (psychologist)	Traditional hierarchical roles are less emphasized, and leadership is potentially more fluid or distributed. It underscores the significant role of nurses and suggests that effective teamwork and coordination are prioritized over formal hierarchical structures
	“I think it’s us [the nurses] who compensate for the professional groups that are not in LEC.” (nurse)	Illustrating the compensatory role of nurses, this quote emphasizes their unique contributions to patient-centered transformations, bridging gaps left by the absence of, i.e., secretaries, occupational or physical therapists
	“I can sometimes feel that I don’t have a fixed position as a nurse in LEC—I think that can be difficult sometimes.” (nurse)	Acknowledging the struggle with role ambiguity, this quote encapsulates the challenges nurses face in defining their roles within the collaborative structure of LEC
	“The nurses are very skilled, but there are still tasks they decline, and then it ends up with me. And I think, ‘That wasn’t a medical task,’ but then I suppose we as doctors feel, ‘Well, I’ll have to do it then.’” (physician)	Challenges related to task distribution within the healthcare team, the skills and limitations of different professionals, and the impact of these dynamics on the physician’s workload and sense of role clarity
	“There are also some things that I’m not necessarily the best at, but which the system has decided I am best suited for, such as sending a referral or writing a status report, or similar tasks.” (physician)	Implies that the system’s decision on task assignments may be based on factors such as role hierarchy, procedural requirements, or resource allocation, rather than the physician’s actual skills or preference

### Transformation Towards Person-Centered Care

With the emergence of LECs, there has been a noticeable shift from a purely medical focus towards a more comprehensive bio-psychosocial approach. This evolution represents a significant departure from traditional healthcare practices towards a more person-centered care that takes into account the multifactorial needs of patients experiencing late effects. Our results underline the critical role healthcare professionals in LECs play in addressing various aspects of patients’ and family caregivers’ lives, often extending beyond the conventional treatment boundaries found in standard cancer clinics. This approach allows for the option of “simply listening,” which challenges the natural inclination of healthcare professionals to take action. From a psychodynamic perspective, this shift requires healthcare providers to engage in deeper self-reflection and emotional attunement, recognizing the unconscious dynamics at play within the therapeutic relationship. The act of “simply listening” can evoke a range of countertransference feelings in healthcare professionals, such as anxiety about not being able to “fix” the problem, which can challenge their professional identities. However, this also presents growth opportunities, allowing them to develop a more nuanced understanding of their patients’ needs, and the possibilities to go into partnership with patients and caregivers empowering them in the healing process. This transformative journey, as highlighted by all three groups of participants, is an ongoing process that significantly impacts the role of healthcare professionals within multidisciplinary LECs, ultimately affecting patients’ quality of life.

The strong commitment to address the multifaceted needs of patients and caregivers is made possible by dedicating more time per patient, creating individualized follow-up plans, and conducting weekly virtual MDT meetings across the five LECs. These meetings allow healthcare professionals to learn from each other through interdisciplinary exchanges of knowledge and experiences, reshaping their professional roles. Describing the unique approach within LECs, the participants captured both the curiosity and uncertainty that healthcare professionals experience as they engage in more person-centered care. This underlines the transformative nature of LECs, where the therapeutic alliance between professionals and patients becomes a central focus, shifting the dynamics of care from traditional hierarchies to more collaborative, empowered partnerships. This partnership approach empowers patients and their caregivers, making them active participants in their healing process, which aligns with psychodynamic principles of fostering autonomy and insight within the therapeutic relationship.

### The Therapeutic Space as a Pre-condition

The participants emphasized the importance of creating a therapeutic space as a prerequisite for the collaboration between patients experiencing late effects and their caregivers. This was particularly a new realization for physicians, who traditionally focused more on medical interventions. Nurses, while recognizing the need for therapeutic conversations, also found this challenging. As a result, psychologists were highly valued for their specific expertise in this field, supporting not only patients and caregivers but also advising colleagues without a psychological background and contributing to the weekly MDT meetings. The therapeutic space within LECs can be seen as a crucial container for the complex emotions and psychological processes that both patients and professionals bring to their interactions. It is important to understand that listening, normalizing, and accepting conditions are important focus in LECs. By not promising the patients anything in advance, physicians might be managing transference, where patients project feelings and expectations from past more traditional healthcare relationships onto the healthcare professional. By refraining from offering immediate solutions and instead encouraging exploration of underlying issues, professionals can help patients with late effects gain deeper insights and safely explore their feelings. This approach aligns with the principles of psychodynamic therapy, which emphasizes the therapeutic relationship as a crucial element in fostering self-understanding and emotional healing.

Additionally, the evolving nature of nursing care within LECs underlines the importance of continuously learning new competencies, highlighting the dynamic and transformative aspects of the nursing role within this therapeutic space. This aligns with psychodynamic concepts of professional growth and the ongoing development of a reflective practice, where nurses adapt to the emotional and psychological needs of their patients. Participants emphasized the change in workflow based on different patient needs and the role of the team in further shaping the patient trajectory. This distinction between LEC patients and traditional patients in cancer treatment suggests a more complex, individualized approach that necessitates intensive and detailed care, reflecting the intricate nature of working with LEC patients.

### Redefined Professional Boundaries

The therapeutic space within LECs presented a need for redefinition of the professional boundaries, emphasizing the inclusion of human competencies as essential for engagement with patients and family caregivers. The participants highlighted their active involvement in therapeutic conversations, recognizing not only the physical but also the psychological and emotional state of LEC patients and their caregivers. However, the delicate balance between forming a human connection and maintaining clinical expertise posed challenges, particularly for nurses who described moments of uncertainty in navigating this balance, and for physicians who were more accustomed to a biomedical approach. From a psychodynamic point of view, this redefinition of boundaries may also involve managing the transference and countertransference dynamics that arise in these therapeutic interactions. The psychologists, feeling more at ease in their “natural habitat” as psychosocial factors are prominent, also experienced the need for competence development within the team. This ongoing learning and adaptation reflect the dynamic and evolving nature of the therapeutic roles within LECs, where each professional’s contributions and personal engagement are vital for patient outcomes, and the professionals showed an expanded understanding of each team member’s field of expertise. However, the generalized nature of the nursing role within LECs also presents challenges, as some of the nurses struggled with role ambiguity, finding themselves taking on compensatory tasks or bridging gaps left by other professionals, i.e., administrative tasks. This reflects the psychodynamic concept of possible role conflicts, where the expectations placed on a professional may clash with their self-perception or professional identity.

Acknowledging that it is difficult to clearly define each professional’s role within the collaborative structure, professional boundaries within LECs need to be more fluid in a LEC. Participants emphasized that while each professional group brings unique strengths to the team and supports one another, no single individual or profession holds exclusive expertise.

### Challenges due to Traditional Hospital Structures

Challenges within hospital structures further impact healthcare professionals, particularly in terms of collaboration and role ambiguity. Participants from LECs revealed a significant shift in healthcare team dynamics from traditional hierarchical models to more collaborative and interdisciplinary approaches. While this shift aims to improve patient care by leveraging the diverse skills of team members, it also introduces new challenges related to power dynamics, role ambiguity, task assignments, and effective communication. The distribution of responsibilities within the team, where the ultimate responsibility for diagnosis and treatment falls on physicians, further complicates the cohesion of the team, as power dynamics and hierarchical structures may strain collaborative efforts.

Physicians and psychologists often have defined clinical roles, while nurses, despite their significant contributions as coordinators for all LEC patients, feel more generalized and sometimes take on compensatory roles, such as administrative tasks. This role ambiguity can lead to feelings of frustration and dissatisfaction, which may be expressed as unconscious defenses against the uncertainty and complexity of their roles. For physicians, their appointed semi-leadership role, described as more fluid, sometimes involving invisible tasks, highlights the challenges of navigating professional identity within the multidisciplinary team. This “pseudo-leadership function” might reflect an internal conflict between the physician’s self-perception as a leader and the realities of their role within the team. The concept of a fluid or distributed leadership model within LECs suggests a shift away from traditional hierarchical roles, where effective teamwork and coordination are prioritized over formal structures. However, this shift also requires careful balancing of leadership and improved strategies for team coordination to prevent the potential strain on team cohesion and ensure the delivery of person-centered care.

## Discussion

The study examined the experiences of 15 healthcare professionals from five Danish LECs from a psychodynamic perspective, shedding light on how these professionals are affected by the unique challenges of providing survivorship care in LECs. The experiences of the professionals reflect a complex interplay of roles, responsibilities, challenges, and uncertainties. While they acknowledged the performance pressure and uncertainty associated with their roles, their experience and curiosity in the therapeutic space contributed to their confidence in navigating these challenges. The study emphasized the need for a nuanced understanding of the evolving role of different professionals in a LEC and the importance of psychosocial person-centered care, which requires continuous learning and adaptation to new competencies.

The theme “Transformation Towards Person-Centered Care” emphasizes addressing late effects comprehensively by integrating diverse aspects of a patient’s everyday life. This approach creates opportunities for patients to express new, often overlooked concerns that traditional cancer care might miss, thereby enhancing person-centered care. Research shows that when patients with cancer feel cared for in a person-centered manner, their physical and psychological symptoms and overall distress decrease [22]. Elkefi et al. [23] further highlight that adopting person-centered strategies within cancer care organizations leads to improved outcomes. In the current study, this approach is consistently applied across healthcare professionals, regardless of their roles. It is recommended that future survivorship care expand this model to fully utilize the varied competencies within LECs. However, achieving this requires an ongoing transformation involving collaboration among healthcare professionals, patients, and caregivers. This approach is already well established in palliative care, where person-centered care is the gold standard [24], offering valuable insights and practical recommendations that could be adapted for managing late effects. Patients dealing with late effects often face unique challenges such as difficulties in the labor market [10] and specific social and lifestyle issues [25] highlighting the need for tailored, person-centered support. However, it is essential for person-centered care in LECs to have a focus on empowering patients and caregivers to take an active role in managing the long-term physical and psychosocial impacts of cancer treatment [26], rather than expecting to be “fixed” without their own involvement. When patients do not participate actively as partners in their own healing, positive progress may be hindered. In this context, healthcare professionals must have the knowledge and skills needed to confidently engage patients in discussions about self-management, which may require additional training [26].

In the study, we found that developing *The Therapeutic Space* was pivotal to accommodate the needs of the patients/cancer survivors and mitigate their late effects. This required nurses and physicians, in particular, to adjust their approach to patient care, adopting a more therapeutic focus. Cancer nurses bring strong skills in building relationships with patients [27], but the therapeutic focus in LECs was challenging. For the majority of traditional follow-up cancer clinics, there are inadequate resources, a lack of time to address the patient’s health-related problems after cancer, and a dominating focus on the detection of relapse of cancer [5]. In the newly established LECs, more time is allocated per patient, which makes it possible to listen to and address all the late effects of the patient and develop the therapeutic space. Acknowledging the necessity of entering a therapeutic space during consultations in the LECs to help the patients transformed and reshaped the clinicians’ professional roles, as they moved away from conventional professional identities. However, several of the professionals found this challenging, and the nurses and physicians had no formal education on how to create this therapeutic space except for their pre-graduate education. Thus, the psychologists were fully aware of their double responsibility towards both patients and colleagues.

Within the therapeutic space of LEC, *professional boundaries were redefined* and more fluid, reflecting an emphasis on human competencies as a cornerstone for engagement. While there is a desire to provide holistic support, there was also a fear of crossing professional boundaries, reflecting the delicate balance between human connection and clinical expertise. Experience and a sense of curiosity seemed to be vital, although moments of uncertainty were present. Palliative care is also an inspiration when it comes to teamwork, where Klarare and colleagues describe communication as the key to working truly interdisciplinary [14], and the changed professional boundaries necessitate new competencies and continuous learning. One could argue that patients’ experiences with cancer care may be positively influenced in settings with a high degree of interdisciplinarity. Accordingly, Tremblay et al. conclude that intensive interdisciplinary teamwork has a positive impact on key aspects of patient-reported quality of cancer care [28].

The redefined boundaries sometimes resulted in *Challenges due to Traditional Hospital Structures*: The LECs faced challenges from entrenched hospital structures, where old roles persist despite differing workflows [29]. These challenges may hinder effective collaboration within the clinical setting. Furthermore, new dynamics emerge, with nurses taking on a role in coordinating patient pathways. Bashkin et al. even suggest that nurses, provided with the proper training and competencies, could be care managers throughout the continuum of cancer care [15].

This shift in power distribution may lead to role ambiguity. In these dynamics, physicians and psychologists have well-defined roles, which can contrast with the more generalized role of nurses. Chan et al. found that physicians felt more confident in delivering survivorship care interventions compared to nurses and allied health professionals [30]. Finding a balance between roles and adapting to changing responsibilities becomes important. There is a recognition that nurses often fill gaps left by other professional groups, emphasizing the importance of coordinated, multidisciplinary care within LEC. This reflects a common challenge in healthcare settings where administrative or procedural roles may not always align with the skills or strengths of medical professionals. It underlines the need for a more nuanced approach to task assignment that considers both individual capabilities and institutional requirements. At the same time, the organization of the new LECs provides a unique opportunity for interprofessional collaboration, which may not exist in other hospital settings with a higher degree of organizational limitations [31].

### Clinical Implications

Improved collaboration across professional roles could provide survivors with more cohesive, supportive care, though traditional hospital hierarchies may still pose challenges. Increased professional development also promises a higher level of tailored, competent care for addressing complex late effects.

### Research Implications

Our findings provide valuable directions for further research and potential improvements in the provision of care to cancer survivors in LECs.

### Strength and Limitations

All Danish LECs were included in the study, and we achieved a high participation rate of 15 out of 20 (75%) healthcare professionals, despite their busy schedules. This high participation ensured a wide range of professional backgrounds and experiences. All participants demonstrated a high level of awareness and self-reflection regarding their professional development, which may have influenced the findings. However, such self-awareness and professional maturity appear to be crucial for delivering high-quality care within a LEC.

Using focus groups proved to be an effective design for uncovering diverse professional perspectives, as they provided a safe and supportive environment for open discussion. We believe we reached data saturation with the sample we had, although only two psychologists were represented. Some LECs employ occupational therapists and social workers. Future research should include these professionals’ perspectives and explore whether the gender of the professionals might influence the findings, as all participants in this study were women.

## Conclusion

This study’s insights into professional dynamics within LECs highlight the need to rethink survivorship care beyond traditional biomedical models. The evolving roles of healthcare professionals underscore the importance of interdisciplinary, person-centered approaches that address both conscious behaviors and unconscious attitudes shaping care delivery. Thus, therapeutic spaces in LECs function not only as settings for patient care but also as reflective environments where health care providers engage with empathy and reassess relational norms. This may have significant implications for professional education, which should include relational and psychological competencies alongside clinical expertise. In line with this, the ESMO Expert Consensus Statements on Cancer Survivorship also emphasize that education is key to improving survivorship care and that training healthcare professionals is essential to meet specific survivorship needs.

The study’s insights also call for expanded research into the psychological and interpersonal dimensions of care in survivorship contexts to promote a development that supports a shift toward more flexible, responsive, and psychologically attuned care models—challenging conventional hospital-based paradigms and informing future research, policy, and practice.

## Supplementary Information

Below is the link to the electronic supplementary material.

Supplementary file1 (DOCX 19 KB)

## Data Availability

The data are with main author and she can be contacted if needed.
