# Supplementary material for: Professional Roles in Danish Clinics for General Late Effects After Cancer—A Qualitative Study
Source: J Cancer Educ. 2025 Jun 6;41(2):302–14. doi: 10.1007/s13187-025-02660-9 (PMC13157456; doi:10.1007/s13187-025-02660-9)
Supplement: Supplementary file 1 — Supplementary file1 (DOCX 19 KB) [file 13187_2025_2660_MOESM1_ESM.docx]

**Supplementary file 1: Semi-structured Interview Guide for Nurses/Physicians/Psychologists:**

- What is your role as a Nurse/Physician/Psychologist in the late effects clinic?
- How would you describe this role compared to other places where you have worked as a Nurse/Physician/Psychologist?
- What specific competencies are particularly utilized?
  - In relation to the patient?
  - In relation to caregivers?
  - In relation to organizational tasks?
  - In relation to development/research?
- Can you provide a case example where your Nurse/Physician/Psychologist expertise played a significant role?
- What do you consider to be the most important prerequisites for being a competent Nurse/Physician/Psychologist in a late effects clinic?
- How can management best support your work?
- What roles do your collaborators have?
- Are the roles well-defined?
- How is your professional expertise influenced by working in a multidisciplinary environment?
- How do you maintain your own professional boundaries?
- How should your competencies develop in the future (wishes/needs)?
- Is there anything you would like to add before we conclude?
